# Measuring health literacy among low literate people: an exploratory feasibility study with the HLS-EU questionnaire

**DOI:** 10.1186/s12889-017-4391-8

**Published:** 2017-05-19

**Authors:** Hannelore Storms, Neree Claes, Bert Aertgeerts, Stephan Van den Broucke

**Affiliations:** 1 Faculty of Medicine and Life Sciences, Hasselt University, Hasselt, Belgium; 2 Campus Diepenbeek, Faculty of Medicine and Life Sciences, Hasselt University, Agoralaan Gebouw D, B-3590 Diepenbeek, Belgium; 3 Academic Center for General Practice, KU Leuven – University of Leuven, Leuven, Belgium; 4 Academic Center for General Practice, Kapucijnenvoer 33 blok j, bus 7001, B-3000 Leuven, Belgium; 5 Psychological Sciences Research Institute, Université catholique de Louvain, Louvain-la-Neuve, Belgium; 6 IPSY - Place Cardinal Mercier 10 bte L3.05.01, B-1348 Louvain-la-Neuve, Belgium

**Keywords:** Health literacy, Patient-centered care, Feasibility, Usability, Vulnerable population

## Abstract

**Background:**

Health literacy (HL) is defined as necessary competencies to make well-informed decisions. As patients’ decision making is a key element of patient-centered health care, insight in patients’ HL might help healthcare professionals to organize their care accordingly. This is particularly true for people in a vulnerable situation, potentially with limited HL, who are, for instance, at greater risk of having limited access to care [1, 2].

As HL correlates with education, instruments should allow inclusion of low literate people. To that end, the relatively new instrument, HLS-EU-Q47, was subjected to a comprehensibility test, its shorter version, HLS-EU-Q16, was not. Therefore, the goal of this study was to examine feasibility of HLS-EU-Q16 (in Dutch) for use in a population of people with low literacy.

**Methods:**

Purposive sampling of adults with low (yearly) income (< €16,965.47) and limited education (maximum high school), with Dutch language proficiency. Exclusion criteria were: psychiatric, neurodegenerative diseases or impairments. To determine suitability (length, comprehension and layout) participants were randomly distributed either HLS-EU-Q16 or a modified version and were interviewed directly afterwards by one researcher. To determine feasibility a qualitative approach was chosen: cognitive interviews were carried out using the verbal probing technique.

**Results:**

Thirteen participants completed HLS-EU-Q16 (*n* = 7) or the modified version (*n* = 6). Questions about ‘disease prevention’ or ‘appraisal’ of information are frequently reported to be incomprehensible. Difficulties are attributed to vocabulary, sentence structure and the decision process (abstraction, distinguishing ‘appraising’ from ‘applying’ information, indecisive on the appropriate response).

**Conclusions:**

HLS-EU-Q16 is a suitable instrument to determine HL in people with limited literacy. However, to facilitate the use and interpretation, some questions would benefit from minor adjustments: by simplifying wording or providing explanatory, contextual information.

**Electronic supplementary material:**

The online version of this article (doi:10.1186/s12889-017-4391-8) contains supplementary material, which is available to authorized users.

## Background

As health care is increasingly becoming more patient-centered, patients are, sometimes reluctantly, expected to assume more responsibility for their care process. To allow them to take up this responsibility, it is important that they have the necessary competencies to take well-informed decisions [3, 4]. In addition to reading and numerical skills applied in a medical context, this also involves more complex and interconnected abilities, such as acting upon written health information, communicating needs to health professionals, understanding health instructions, applying them correctly to their personal situations, and taking action if needed [5].

These competencies are contained in the concept of health literacy (HL), which can be defined as a person’s knowledge, motivation and competencies to access, understand, appraise, and apply health information in order to make judgments and take decisions concerning health [6]. The importance of HL for health care services and systems is recognized worldwide, and its reach is increasing: while originally the concept was mainly used within health care services, in recent years it is also gaining grounds in public health [3, 7]. A growing number of studies show that people with low HL not only have a lower adherence to medication, poor self-care and worse treatment outcomes, but are also less likely to engage in health promoting behaviour, participate in screening programs, or make use of preventive services [8–11].

Over the past years, a range of HL measurement tools have been developed, which vary in their approach, design, and purpose [7, 12]. Some tools focus on HL related to specific conditions, such as diabetes, cardiovascular disease, cancer, oral health, or mental health, or on specific population groups, while others are more generic. Some have been developed for the purpose of screening functional HL problems in clinical settings, and are consequently short and easy to use, while others provide an in-depth assessment of HL and its dimensions at population level, using a broader approach [12]. Examples of the first kind of tools are the Rapid Estimate of Adult Literacy in Medicine (REALM) [13, 14], the Newest Vital Sign (NVS) [15], and the Short Assessment of Health Literacy (SAHL) [16]. Examples of the second kind are the Critical Health Competence Test (CHC) [17], the Swiss Health Literacy Survey [13, 18], the Health Literacy Management Scale (HeLMS) [19] and the Health Literacy Questionnaire (HLQ) [20] .

A relatively new measure, which can be used both for screening and for a more in-depth investigation of HL, is the European Health Literacy Survey (HLS-EU-Q). A consortium of European organisations developed this instrument guided by a conceptual model of HL derived from a systematic literature review [7]. The original tool (HLS-EU-Q47) consists of 47 items addressing self-reported difficulties in accessing, understanding, appraising and applying information in tasks concerning decision-making in health care, disease prevention, and health promotion, and was designed for face-to-face or telephone interviewing [21]. A short form (HLS-EU-Q16), comprising 16 of the 47 items, was developed for quicker screening of HL, either through interview or via self-report (pen and paper or online) [22]. Its validation was done via Rasch modelling (1-parametric dichotomous model) and item selection on the basis of content and face validity (item relevance) [22–24]. Although no statements regarding sub-dimensions of HL are allowed, HLS-EU-Q16 is a good approximation of the 47-item version, with a high correlation (*r* = .82) with the general HL score of the HLS-EU-Q47, and a 75.8% concurrent classification of respondents as having insufficient, limited and sufficient HL. HLS-EU-Q16 has been used in several countries, including Belgium [25], the Netherlands [16, 26] and Germany [27, 28].

A major advantage of short self-report measures of health literacy such as HLS-EU-Q16 is that they are relatively easy to administer, both in a clinical setting and at population level, while allowing a comparison with other (patient or general) populations. Some scholars have also used online versions of the tool [25], which is particularly useful for population research involving large samples. However, the downside is that completing a self-report questionnaire requires a certain level of literacy from the respondents. Research by Van der Heide et al. using the HLS-EU-Q47 revealed that limited HL is frequently reported in those with lower levels of education [21, 29]. To allow the inclusion of low literate persons in the survey, the original HLS-EU-Q47 underwent a comprehensibility test for all languages involved, but a similar test was not performed on the short self-report version [21]. Given the difference between a computer-assisted interview and a self-report questionnaire, it is important to ascertain that HLS-EU-Q16 can also reach and detect people with low literacy, as an instrument that is judged to be comprehensive and easy to execute enhances the participation of people and the quality of the measurement.

The aim of this study was to examine the suitability of HLS-EU-Q16 for use in a population of people with low literacy in vulnerable conditions. As this group of people can be expected to have limited health literacy, it is of paramount importance to measure their health literacy adequately. The validation of HLS-EU-Q16 will not be discussed in this research, as it is documented elsewhere.

## Methods

To verify the suitability of HLS-EU-Q16 with low literate persons, a feasibility study was performed using a qualitative approach. In this study, the comprehensibility of HLS-EU-Q16 (in Dutch) and of a modified version of the instrument was tested with a group of people with potentially low (health) literacy on account of their education level.

### Development of a modified version: HLS-EU-Q16-EZ

To develop a modified version of HLS-EU-Q16, one researcher (HS) examined HLS-EU-Q16 on sentence structure and language. Level 'B1' as defined in the Common European Framework of Reference for Languages was used as a reference, because documents of this level are supposed to be understood by 95% of the population speaking that language [30]. This preliminary analysis was carried out in an intuitive manner, using http://www.zoekeenvoudigewoorden.nl to look up words on B1-level and http://www.accessibility.nl/kennisbank/tools/leesniveau-tool to verify sentence structures. Subsequently, an expert organization on accessible communication (non-profit organization 'Wablieft vzw') was tasked to independently develop an alternative version of the questionnaire, called HLS-EU-Q16-EZ. Questions assessed as too complex (use of jargon or difficult words, subordinate clauses) according to expert organization’s opinion, were rewritten in lay-language and everyday speech. Moreover, the expert organization was instructed to only modify if necessary and, if adjustments were made, to rewrite them with a maximum resemblance to the original formulation.

### Selection

Purposive sampling was used to select participants for the study. Inclusion criteria were: being aged between 18 and 70 years, a low level of education (preferably primary school, highest being high school because of the compulsory schooling in Belgium until the age of 18) and low income (defined as a gross family income in the previous year ≤16,965.47 euro for the applicant, raised with 3140.77 per person living on the same address; or receiving a specific reimbursement designated for people who are considered to be in a ‘vulnerable’ position). To illustrate, low income requirements in 2014 were set at maximum 16,743.70 euro in the previous year for the applicant (and 3099.72 per additional person), while an average gross income then amounted 46,197 euro [31–33]. To participate, being able to read and understand Dutch was a language requirement, although it was not necessary to be a native speaker. Exclusion criteria were: having a psychiatric and/or neurodegenerative disorders (e.g., Alzheimer’s, Parkinson, ...); (being formally recognized as) having physical, intellectual or sensory impairment(s); or not being able to interact with the researcher. To recruit people eligible for participation, 11 organizations working with people in a vulnerable position were contacted, which is a methodology similar to the ones used in previous research with vulnerable groups [34, 35]. The number of eligible participants was difficult to determine beforehand: recruitment was scheduled on informal gatherings with fluctuating number of people showing up. Saturation in participants’ opinions was pursuit, rather than a predetermined sample size, as a consequence of challenging recruitment.

### Data collection

To determine the suitability of HLS-EU-Q16, cognitive interviews about the questionnaire were performed by one researcher using the Verbal Probing technique [36]. The suitability of the questionnaire was operationalized as participants’ opinion about: the length of the questionnaire and questions; comprehension of questions and language; clarity of instructions; the response process; the layout of the questionnaire and any other remarks not directly addressed by the researcher.

Participants randomly received either HLS-EU-Q16 or HLS-EU-Q16-EZ, dividing them in two groups. To avoid stigmatization, everybody who was present at the meeting when the interviews were scheduled to be held, could complete the questionnaires that had been randomly distributed to the group, but only those meeting the inclusion criteria were interviewed directly afterwards. The purpose of the study was explained, participants were assured of the anonymity of the study and of the fact that they would not be judged, which stimulated them to give their honest opinion about the questionnaire. Information was gathered through a semi-structured interview, carried out by the same researcher. Interviews were audio-taped to facilitate analysis afterwards. Interviews were ended because of data saturation.

#### Cognitive interview

Exemplary probing questions to assess comprehension, were: “What does [term] mean to you?” “Can you explain [question] in your own words?”, “Was that [question] easy or hard to answer?”. When questions or words were unclear, the corresponding question of the other version was presented (Appendix 1). To determine the layout of the questionnaire, participants were asked to choose the most preferred one out of six exemplary layouts (Additional file 1).

#### Statistical analysis

Out of interest and in order to determine over- or underestimation of health literacy levels, non-responses and health literacy scores were statistically analyzed. HL-scores were calculated by coding responses “very easy” and “easy” as 1; “difficult” and “very difficult” as 0, subsequently summing them, which results in a score between 0 and 16, categorized as inadequate (<9), limited (9–12) or sufficient (13–16) health literacy [7, 23]. Sociodemographic characteristics (gender, age, level of education, income) were determined. Data analysis was performed using SPSS 22.0.

## Results

A total of 13 participants from three organizations were included in the study which took place from October 2015 until December 2015. Three respondents eligible for participation did not consent to interviewing and were excluded. Nine people were excluded from the study because they did not meet inclusion criteria. The characteristics of the participants are shown in Table 1. The majority of participants was female. Respondents’ age was on average 59 years old with SD 8.

**Table 1:** Characteristics of participants

	HLS-EU-Q16	HLS-EU-Q16-EZ
Participation	7	6
Gender		
male	2	2
female	5	4
Age		
< 40	0	0
40–50	2	0
51–70	5	6
Education		
None	1	2
Primary school	4	2
High school	2	2
Income		
Low family income	7	6
High reimbursement	1	2

### Informal test on HLS-EU-Q16

To construct a questionnaire on B1-level, at least 12 items would have needed to be revised based on vocabulary and/or sentence structure (Q1, Q4 – Q13, Q16).

### Cognitive interview

Difficulties in completing the questionnaires (HLS-EU-Q16 = A; HLS-EU-Q16-EZ = B) as reported by participants in the interviews, are summarized in Table 2. Words that were unclear in A-version were ‘preventive’ (*Dutch ‘preventief’*) (Q10), ‘mental wellbeing’ (*Dutch ‘geestelijk welzijn’*) (Q13), ‘to judge’ (*Dutch ‘beoordelen’*) (Q5 and Q11) and ‘media’ (Q12).

**Table 2:** Problems concerning the questionnaires HLS-EU-Q16 (= A) and HLS-EU-Q16-EZ (= B)

Problem	Item - Questionnaire	Quote
Comprehension problems due to…		
Vocabulary	▪ Questionnaire A	▪ “The questionnaire should be in everyday speech.” (respondent 3, respondent 8)
	▪ Questionnaire A	▪ “I think you should make it simpler, a lot of people will not understand [*the questionnaire (A)*]. If even I find it difficult..” (respondent 9)
	▪ Q5 -A and Q11 - A	▪ “What does the word ‘to judge’ (Dutch ‘beoordelen’) mean?” (respondent 3, respondent 9, respondent 11, respondent 12)
	▪ Q9 - A	▪ “Drinking too much: I should drink more water. You mean too much water?” (respondent 5)
Sentence structure	▪ Q11 - B	▪ “I have had to read the question [*Q11 (B)*] several times in order to understand. Sentences should be shorter.” (respondent 2)
	▪ Questionnaire A	▪ “The questions should be shorter.” (respondent 8)
Decision process problems due to…		
Referencing to general practitioner	▪ Q11 - A	▪ “What does the media have to do with it? If my general practitioner has something to say to me, he should say that to my face” (respondent 3)
	▪ Q1 - B	▪ “Q1 (B) is not applicable to my situation: my general practitioner properly informs me, so I would want an extra answer option ‘not necessary’ because I get all my information from my general practitioner” (respondent 6)
	▪ Q5 – B	▪ “I understand the question and the words, but I still do not know what to answer. I rely on my general practitioner; he is the one I can trust…so I do not know what to answer” (respondent 2)
	▪ Q8 - A	▪ “That is also a difficult question [*Q8(A)*], if you have mental health problems, you talk it through with your general practitioner; if you are depressed, you must go to the hospital, so I would talk to my general practitioner” (respondent 9)
	▪ Q11 - A	▪ “What does the media have to do with it? If my general practitioner has something to say to me, he should say that to my face” (respondent 3)
Cannot relate to questions about the media	▪ Q11 - A	▪ “What does the media have to do with it? If my general practitioner has something to say to me, he should say that to my face” (respondent 3)
	▪ Q11 - A and Q12 – A	▪ “I follow the media, but I don’t always agree with them, it is difficult to answer” (respondent 9)
	▪ Q15 - A	▪ “I don’t understand what they mean ‘to understand information presented by the media about being healthy’?” (respondent 10)
Different interpretations of preventive examinations: confusion if question is restricted to cancer screening	▪ Q10 – A	▪ “I have renal failure, so I am subjected to many tests, I always have to get myself checked out, so I am examined preventively.” (respondent 6)
	▪ Q10 - A	▪ “What do you mean with preventive? I have osteoporosis, that doesn’t happen in 1 day” interviewer refers to preventive screening of cancer “I am doing all of them [*cancer screening*] so that is easy for me” (respondent 9)
Difficulties distinguishing between “appraising” or “applying”	▪ Q5 - A	▪ “I don’t know what the question is about. I think it is about judging the second doctor or is it about judging if you need a second opinion? I answered as if I were already there [*with the second doctor*]. You can make a question like ‘Do you find it easy to judge if u need another advice?” (respondent 8)
Unclear distinction between questions	▪ Q1 - A vs Q6 - A	▪ “What is the difference between Q1 (A) and Q6 (A)?” (respondent 8)
	▪ Q3 - A vs Q4 - A	▪ About unclear distinction between Q3 (A) and Q4 (A) “You can change Q4 (A) to ‘Are you capable of taking your medication independently?’.” (respondent 8)
Irrelevance of questions in specific situations		
	▪ Questionnaire B	▪ “The questionnaire is rather difficult, some questions I am not able to answer because I do not find myself to be in the situation that is being portrayed.” (respondent 6)

#### Comprehension

Despite vocabulary and sentence structure being unclear, participants reported difficulties related to their decision processes: being confused about which answer option corresponded to their thoughts on the question. Overall, problems related to comprehension can be categorized as:

- 1° Comprehension problems due to
  - vocabulary and
  - sentence structure
- 2° Decision process, often abstraction problems:
  - Referencing to general practitioner
  - Not relating to questions about the media
  - Different interpretations of preventive examinations: confusion if question is restricted to cancer screening
  - Difficulties distinguishing between 'appraising' or 'applying' - questions
  - Unclear distinction between questions
- 3° Irrelevance of questions

The least understood in both questionnaires are questions 5, 10 and 11. Questions 1, 3 and 6 were perceived as more comprehensible in the B-version.

#### Length, response process and layout

All respondents were satisfied with the length of the questionnaires. Few remarks were made with regard to the response process *(“Answer options should not be ‘rather’ (Dutch ‘tamelijk’) easy or difficult, but ‘less’ (Dutch: ‘minder’) or ‘not quite’ (Dutch ‘niet zo’)”* *questionnaire A, respondent 8*) and layout (“*Important and/or repetitive sentences should be typed bold. I want space to write remarks and there should be more spacing between questions.”* *q* *uestionnaire A, respondent 6*). With regard to layout, the majority of participants agreed that the questionnaire had to be structured, with borders separating different questions and spacing between them. Important (parts of) sentences, like the repetitive basic question, should be typed in bold. Some participants preferred being able to write down comments supplementary to each question. When presented with 6 exemplary layouts (Additional file 1) two groups of respondents can be distinguished: one that preferred examples 1 and 6, and the other preferring examples 2 and 5. Presumably, the former are similar because of spacing, the latter because of the only difference being the (lack of) boarding. There is a slight preference for empty boxes to check (example 1), instead of a repetition of each answer option (examples 2, 5 and 6). Almost all participants preferred repetitive questions or important parts of the question being typed bold.

### Missing responses

Non-responses were analyzed quantitatively. In Table 3 missing responses are illustrated. The sum of non-responses generated in the group completing the HLS-EU-Q16 was higher (*n* = 17) than the number of total non-responses for those who filled out the HLS-EU-Q16-EZ (*n* = 10). Incomplete questionnaires were generated by 7 of 13 participants, of which four filled out the HLS-EU-Q16; one participant completed only half of HLS-EU-Q16.

**Table 3:** Missing responses in HLS-EU-Q16 and HLS-EU-Q16-EZ

	HLS-EU-Q16 (*n* = 7)	HLS-EU-Q16-EZ (*n* = 6)
Sum of non-responses	17	10
Participants	4	3
Missing responses per participant		
8	1	0
6	1	1
3	0	1
2	1	0
1	1	1
Question with missing responses		
Q5	2	2
Q1; Q12; Q14	2	1
Q3; Q10; Q11; Q15	1	1
Q2; Q6; Q8; Q9; Q13	1	0
Q4	0	1

## Discussion

When developing questionnaires to measure HL, it is important to consider respondents’ level of education. Therefore, feasibility of a HL-instrument (HLS-EU-Q16) was examined in people with low literacy. The objective was to determine how respondents experienced length, comprehension and layout (overall: ‘suitability’) of this instrument. In this feasibility assessment, a variety of factors (difficulty of a text, layout...) can be addressed. Moreover, this is a subjective approach, as opposed to – in literature sometimes contested – readability tests in which texts are quantitatively assessed [37]. Moreover, interviewing the intended users of the questionnaire is helpful to gather in-depth information [38].

The results of this study indicate that HLS-EU-Q16 is a questionnaire suitable to determine the level of HL in a population of people of low literacy on account of their lower income and education. Overall, the questions of the instrument are well understood. Moreover, cognitive interviews regarding participants’ opinion about the questionnaire revealed that less items of HLS-EU-Q16 were perceived to be difficult or required a revision than suggested by the researcher’s informal test on B1-level for documents. Based on presented findings it can be concluded that changing the vocabulary is insufficient to improve the suitability of HLS-EU-Q16.

### Reported difficulties

When referring to the initial framework of HLS-EU questionnaire [3], questions often reported to be less comprehensive were those about ‘disease prevention’ (domain) or ‘appraisal’ of information (competency). Indifferent of domain or competency, all questions referring to a potential role of the media in health care seemed hard to relate to. Referring to one’s general practitioner as a source of health information was an apparent and frequently mentioned response. This statement was most often accompanied by the comment that respondents had difficulties deciding how to respond. Indirectly, these findings revealed the trust in general practitioners and consequently their crucial position in health care, particularly for vulnerable groups (people with low levels of income and education) [39–41].

Questions the least understood in both questionnaires resulted in high number of non-responses. Although reliability of HL-scores is irrelevant because of the research design, non-responses illustrated limited comprehension of HLS-EU-Q16. However, respondents reporting incomprehension of this instrument most often answered at least half of the items. Hampered comprehension of questions could be attributed to converging difficulties: vocabulary that was perceived as difficult (‘to judge’ *(Dutch ‘beoordelen)*; ‘media’), abstraction, as well as participants being indecisive on the appropriate response.

Some difficulties persisted even when HSL-EU-Q16-EZ (the modified questionnaire) was presented to participants. Because data collection with HLS-EU-Q16 was not carried out face-to-face, as opposed to HLS-EU-Q47 in which Computer Assisted Personal Interviewing (CAPI) was used, people of low literacy groups could benefit ​from a minor adjusted HLS-EU-Q16 [21]. To resolve both comprehension and decision process problems, some questions would benefit from an additional explanation. Providing extra information in short, explanatory sentences can bypass problems concerning the meaning of words. Moreover, it may rule out confusion amongst respondents by giving more context and making it easier for people of low literacy groups to respond. The suggestion for contextual information is made based on reports of confounding questions: some questions were perceived as too similar, leaving respondents confused about their objective. To situate questions, providing information with regard to the ‘competencies’ to which questions are referring to (for instance, distinguishing questions about ‘appraising’ from those about ‘applying’) could facilitate interpretation. In conclusion, it is advisable to add information similar to the information that would be provided by the interviewer when collecting data based on CAPI (HLS-EU-Q47). Although occurring rarely, questions can truly be irrelevant because of very specific situations: for instance, someone who needs to rely on health care professionals’ (or informal caregivers’) decision because of their medical condition, might find some questions irrelevant; someone with no or limited access to 'the media'. In these rare cases, people would benefit from an additional category to report accordingly; adversely, it increases the likelihood of non-responses. When the questionnaire would be used with forced answering (electronically), benefits and disadvantages of an extra category should be outweighed.

The following changes might improve the understanding of the questions: basic instructions can allude on how questions should be interpreted, guidance on answering options can be provided in case people would feel to only ‘rely on their general practitioner or other (non-)medically trained people’; questions can be broken down into different components, so it can be avoided that people find the portrayed situation in a part of the question irrelevant to them; the meaning of terminology might be explained through an example, for instance of a preventive examination, or questions might be put into a different, more clarifying order (per domain or competency surveyed) [42]. These suggestions should be implemented cautiously, avoiding narrowing the questionnaire to specific health-related topics. However, some additional information might increase comprehensibility and potentially response rate.

### Implications

In this research, the focus was on HLS-EU-Q16 (in Dutch). Because HLS-EU questionnaires are available in different languages, but not all of them were tested on ‘cultural applicability’, it would be helpful to determine feasibility of HLS-EU questionnaire beforehand, preferably by surveying or interviewing the intended users [7, 38, 43]. Evaluating the questionnaire’s suitability and eventually readjusting it, might be particularly useful if questionnaires are intended to be used in a paper-pen version, without assistance from a researcher [44]. Because assessing feasibility is subjective, adjustments made in correspondence with respondents’ feedback are specific for those interviewees and therefore not generalizable.

### Strengths and limitations

A strength of this study is the insight that was gained with regard to the cognitive process of respondents when completing HLS-EU-Q16. Moreover, this study was specifically targeting a population considered to be vulnerable because of low levels of education and income. To the best of our knowledge, this was the first study targeting this population to examine feasibility of a questionnaire. Similar studies have been carried out in a peadiatric setting or targeting vulnerable groups, such as refugees. [43, 45]. The research of Wångdahl et al. examining HL in refugees, indicated sufficient language proficiency – preferably respondents’ mother tongue – and modification of questions or answering options to be contributing to questionnaires’ accessibility. In their research, HLS-EU-Q16 (in Swedish) was modified based on data collected through cognitive interviews [43].

The main limitations of this study are related to the vulnerable character of the population. Recruitment was challenging: an unusual, yet inclusive approach was used to reach eligible participants in order to avoid stigmatization. Moreover, reaching people of younger age meeting the education requirement seemed even more challenging, which was to be expected given the compulsory education until the age of 18 years old in Belgium. Despite efforts to reach this population, it still resulted in relatively small sample size, but as testing suitability is a qualitative approach, it is not based on representative samples. Data saturation was reached when 13 participants were interviewed, a sample regarded as sufficient [46].

Although a test-retest design would have allowed to generate interesting data, reproducibility was not feasible. Participants seemed to find it difficult to distinguish between answering the 16 questions and answering on the feasibility of these questions (interviewer’s asking about how the questionnaire was perceived often resulted in respondents reporting about their answers). Asking them to participate in a second, similar survey would probably lead to more confusion.

Interviews were conducted using the Verbal Probing technique: the researcher attempted to minimize the use of ‘leading’-probes to reduce bias. However, as the purpose of this interview was to analyze comprehension of questions, an example or clarifying sentence was provided, with the intent to get insight in the difficulties reported by participants and potential solutions, but never from the start. Ideally, the interview would have been conducted during the completion of the questionnaire. However, the time between completing the questionnaire and the interview was minimalized to 1 h maximum. A final limitation is that this research was carried out with paper-based questionnaires only. The findings presented in this study are not necessarily transferable to the same instrument in a web-based version. Working with digital surveys requires a specific set of skills: these were not taken into account in this research.

### Future research

Based on reported difficulties, an altered HLS-EU-Q16 can be developed to determine HL in people with low literacy. Based on the findings of this research, some people with potential limited HL might benefit from some questions being formulated more clearly, for instance by adapting wording or by providing contextual information to facilitate interpretation [42]. Moreover, a complementary, modified questionnaire could be provided or it might be useful to determine HL through interviews as opposed to surveying with pen and paper [47].

## Conclusion

Determining HL in people with limited literacy based on HLS-EU-Q16 is feasible. However, to facilitate the use and interpretation, some questions would benefit from minor adjustments: by simplifying wording or providing explanatory, contextual information.

### Additional files

Additional file 1: Exemplary layout. (PDF 146 kb).

## Appendix 1

### Semi-structured interview

#### At the start of the interview

People are reassured to give their own opinion and that they will never be judged because there are no correct or false answers.

The interview is audio-taped: recordings will only be used for the purpose of data-analysis. Anonymity is ensured.

#### Questions

- **1° How did you find the** **questionnaire** **?**
  - In your opinion, what was the questionnaire about?
  - What did you think about:
    - ◦ the number of questions?
    - ◦ the layout of the questionnaire?
    - ◦ the introduction at the beginning of the questionnaire
  - There is no mentioning on how to mark your answers, what was your opinion on this topic?
  - Which improvements would you suggest?
- **2° How did you find the** **questions** **?**
  - What was your opinion on the length of the questions?
  - Was it clear to you what every question was on about?
    - ◦ When not clear: which question(s) was(were) not clear?
      Interviewer provides same question from other version of questionnaire: Is this question [other version of questionnaire] clearer than the original question?
  - Can you explain [question] in your own words?
- **3° How did you find the** **vocabulary** **?**
  - Did you understand all the words used in the questionnaire?
  - Were there any words that were not clear to you?
  - What does the [term] mean to you?
- **4° How did you find the** **answering options** **?**
  - Was that [question] easy or hard to answer?
  - If the question was clear to you, was it clear to you which answering option corresponded with you answer?
  - In your opinion, was it easy or hard to respond in this manner, with the given answering options?
  - Would you have preferred a mentioning on how answering options should be marked?
- **5° Exemplary layouts**
  The interviewer distributes exemplary layouts and explains the differences between them: Which one of these layouts would you prefer and why?
- **6° Suggestions and other remarks**
  Do you have any final remarks or suggestions? There might be something that hasn’t been mentioned in the interview and that you would like to add because it is important to you in order to improve the questionnaire?
