# Supplementary material for: Measuring health literacy among low literate people: an exploratory feasibility study with the HLS-EU questionnaire
Source: BMC Public Health. 2017 May 19;17:475. doi: 10.1186/s12889-017-4391-8 (PMC5438531; doi:10.1186/s12889-017-4391-8)
Supplement: Supplementary file 1 — Exemplary layout. (PDF 146 kb). [file 12889_2017_4391_MOESM1_ESM.pdf]

Additional file 1: Exemplary layout

Example 1

		Very easy	Easy	Difficult	Very Difficult
**1.**	**On a scale from very easy to very difficult, how easy would you say it is to** find information on treatments of illnesses that concern you?				
**2.**	**On a scale from very easy to very difficult, how easy would you say it is to** find out where to get professional help when you are ill?				
**3.**	**On a scale from very easy to very difficult, how easy would you say it is to** understand what your doctor says to you?				
**4.**	**On a scale from very easy to very difficult, how easy would you say it is to** understand your doctor's or pharmacist's instruction on how to take a prescribed medicine?				

Example 2

**1.**	**On a scale from very easy to very difficult, how easy would you say it is to** find information on treatments of illnesses that concern you?	**Very easy**	**Easy**	**Difficult**	**Very Difficult**
**2.**	**On a scale from very easy to very difficult, how easy would you say it is to** find out where to get professional help when you are ill?	**Very easy**	**Easy**	**Difficult**	**Very Difficult**
**3.**	**On a scale from very easy to very difficult, how easy would you say it is to** understand what your doctor says to you?	**Very easy**	**Easy**	**Difficult**	**Very Difficult**
**4.**	**On a scale from very easy to very difficult, how easy would you say it is to** understand your doctor's or pharmacist's instruction on how to take a prescribed medicine?	**Very easy**	**Easy**	**Difficult**	**Very Difficult**

Example 3

		Very easy	Easy	Difficult	Very Difficult
**1.**	On a scale from very easy to very difficult, how easy would you say it is to find information on treatments of illnesses that concern you?				
**2.**	On a scale from very easy to very difficult, how easy would you say it is to find out where to get professional help when you are ill?				
**3.**	On a scale from very easy to very difficult, how easy would you say it is to understand what your doctor says to you?				
**4.**	On a scale from very easy to very difficult, how easy would you say it is to understand your doctor’s or pharmacist’s instruction on how to take a prescribed medicine?				

Example 4

**1.**	On a scale from very easy to very difficult, how easy would you say it is to find information on treatments of illnesses that concern you?	**Very easy**	**Easy**	**Difficult**	**Very Difficult**
**2.**	On a scale from very easy to very difficult, how easy would you say it is to find out where to get professional help when you are ill?	**Very easy**	**Easy**	**Difficult**	**Very Difficult**
**3.**	On a scale from very easy to very difficult, how easy would you say it is to understand what your doctor says to you?	**Very easy**	**Easy**	**Difficult**	**Very Difficult**
**4.**	On a scale from very easy to very difficult, how easy would you say it is to understand your doctor's or pharmacist's instruction on how to take a prescribed medicine?	**Very easy**	**Easy**	**Difficult**	**Very Difficult**

Example 5

1. **On a scale from very easy to very difficult, how easy would you say it is to** find information on treatments of illnesses that concern you?	**Very easy**	**Easy**	**Difficult**	**Very Difficult**
2. **On a scale from very easy to very difficult, how easy would you say it is to** een beslissing over uw ziekte te nemen met behulp van de informatie die de arts geeft?	**Very easy**	**Easy**	**Difficult**	**Very Difficult**
3. **On a scale from very easy to very difficult, how easy would you say it is to** find out where to get professional help when you are ill?	**Very easy**	**Easy**	**Difficult**	**Very Difficult**
4. **On a scale from very easy to very difficult, how easy would you say it is to** understand your doctor's or pharmacist's instruction on how to take a prescribed medicine?	**Very easy**	**Easy**	**Difficult**	**Very Difficult**

Example 6

1. **On a scale from very easy to very difficult, how easy would you say it is to find information on treatments of illnesses that concern you?** Very easy Easy Difficult Very difficult

2. **On a scale from very easy to very difficult, how easy would you say it is to find out where to get professional help when you are ill?** Very easy Easy Difficult Very difficult

3. **On a scale from very easy to very difficult, how easy would you say it is to understand what your doctor says to you?**

   Very easy Easy Difficult Very difficult

4. **On a scale from very easy to very difficult, how easy would you say it is to understand your doctor's or pharmacist's instruction on how to take a prescribed medicine?**

   Very easy Easy Difficult Very difficult
